# Epidemiology and clinical features of rotavirus and norovirus infection among children in Ji’nan, China

**DOI:** 10.1186/1743-422X-10-302

**Published:** 2013-10-08

**Authors:** Lintao Sai, Jintang Sun, Lihua Shao, Shuai Chen, Haihong Liu, Lixian Ma

**Affiliations:** 1 Department of Infectious Diseases, Qilu Hospital, Shandong University, Wenhua Xi Road 107, Ji’nan 250012, Shandong Province, China; 2 Institute of Basic Medical Sciences, Qilu Hospital, Shandong University, Wenhua Xi Road 107, Ji’nan 250012, Shandong Province, China; 3 Department of Laboratory Sciences, School of Public Health, Shandong University, Wenhua Xi Road 44, Ji’nan 250012, Shandong Province, China; 4 Qilu Children’s Hospital of Shandong University, Jingshi Road 430, Ji’nan 250012, Shandong Province, China

**Keywords:** Rotavirus, Norovirus, Acute gastroenteritis, Epidemiology, Clinical symptom

## Abstract

**Background:**

Acute gastroenteritis caused by bacteria, virus and parasite is an important cause of childhood morbidity and mortality in developing countries. Rotavirus and norovirus have been recognized as the most common pathogens causing acute gastroenteritis among children. However, there is still no valuable data about infections of rotavirus and norovirus in children in Ji’nan, an eastern city in China. The aims of the present study are to determine the incidence of rotavirus and norovirus associated acute gastroenteritis in Ji’nan among children, to characterize rotavirus and norovirus strains circulating during this period; and to provide useful epidemiological and clinical data.

**Methods:**

Fecal specimens and clinical data were collected from 767 children (502 outpatients and 265 inpatients) under 5 years of age with acute diarrhea at Shandong University Qilu Hospital and Qilu children’s Hospital in Ji’nan, China between February 2011 and January 2012. Virus RNA was extracted, amplified, electrophoresed, sequenced and phylogenetically analyzed to determine the prevalent genotypes. Chi-square and *U* test were used to compare characteristics of clinical manifestation in each group.

**Results:**

Of the 767 specimens 263 (34.3%) were positive for rotavirus and 80 (10.4%) were positive for norovirus. Among 263 rotavirus positive cases, G3 (40.7%) was the most prevalent serotype, P[8] (46.8%) was the dominant genotype and G3P[8] (31.9%) was the most common combination. All of the norovirus strains belonged to GII genogroup including GII.3, GII.4 and GII.6, of which GII.4 (61.2%) was the predominant genotype. Phylogenetic analysis of the GII.4 sequences showed that 18 GII.4 strains belonged to GII.4 2004–2006 cluster and 31 GII.4 strains were divided into GII.4 2006b cluster. A peak number of rotavirus infections was observed during the cold season from November to next January. Higher rates of norovirus infections were detected from September to November. Most patients with rotavirus and norovirus associated diarrhea experienced vomiting (88.2% and 67.5%, respectively) and fever (79.1% and 46.3%, respectively).

**Conclusions:**

The present study showed that rotavirus and norovirus were still the important causative agents of pediatric diarrhea in Ji’nan during this period.

## Background

Acute gastroenteritis caused by bacteria, virus and parasite is important cause of childhood morbidity and mortality in developing countries [1,2]. Cumulative data from previous studies shows that enteric viruses have replaced bacteria as the most significant pathogen of acute diarrhea [3]. Of these viruses, rotavirus and norovirus have been recognized as the most common etiological agents of pediatric acute gastroenteritis [4-7].

Rotavirus is a double stranded RNA virus and belongs to the family of *Reoviridae* that includes seven serogroups (A-G). Group A rotavirus predominantly results in severe acute diarrhea in children. The genome consists of 11 segments enclosed in a triple layered capsid protein. The outer capsid is composed of two independent neutralization antigens VP4 and VP7. The VP7 is named G-serotype and at least 16 G-serotypes have been recognized to date. The VP4 determines P-genotype and no less than 27 P-genotypes have been reported [8-10]. Based on previous studies, rotavirus infections caused 25 million clinical visits, 2 million hospital admissions and about 611,000 deaths annually worldwide in children, mainly in developing countries [11,12]. Safe and effective vaccine is the most effective tool in preventing the transmission of rotavirus infection. There is only one local rotavirus vaccine licensed for gastroenteritis prevention (group A rotavirus) among children in China. The rotavirus vaccine is not included in the national immunization programs and is relatively expensive in China. Therefore, the coverage of the vaccine is low which leaves most children vulnerable to rotavirus infection. Several epidemiological surveys undertaken in China showed that the infection rates of rotavirus ranged from 28% to 65% [13,14]. However, there is no data associated with rotavirus infection in Ji’nan, an eastern city with a population of about 7 million.

Norovirus classified into the family of *Caliciviridae* has a single-strand, positive-sense, polyadenylated RNA genome that contains three open reading frames (ORFs). ORF1 encodes non-structural proteins including the RNA-dependent RNA polymerase. ORF2 and ORF3 encode a single major and a minor capsid protein, respectively [15]. Based on the partial sequence of the genome RNA, noroviruses are divided into five genogroups (GI-GV) and only GI, GII and GIV infect humans, of which GI and GII can be further divided into at least 8 and 17 genotypes, respectively [16-18]. Noroviruses have been recognized as another most common causative agent causing acute gastroenteritis in children [5-7]. The genome of norovirus is easy to recombine or mutate, and new variants can emerge every several years to become the dominant strains in a certain period [19-21]. Therefore, norovirus infections are difficult to prevent and control.

Some rotavirus and norovirus associated pediatric diarrhea studies had been performed in other cities in China. However, most surveys only focused on the epidemiological feature. This study not only described the epidemiological features of rotavirus and norovirus infections but also compared the clinical peculiarity and severity of symptoms between rotavirus and norovirus. The aims of the present study are (1) to determine the incidence of rotavirus and norovirus associated acute gastroenteritis in Ji’nan; (2) to characterize rotavirus and norovirus strains circulating during this period; (3) to provide useful epidemiological and clinical data which may help for the development of vaccines and treatments.

## Results

From February 2011 to January 2012, a total of 767stool samples were collected from 502 outpatients and 265 inpatients to test for the presence of rotavirus and norovirus. The overall detection rates of rotavirus and norovirus were 34.3% and 10.4%, respectively (Table 1). The results detected by Elisa kit were consistent with the observation by RT-PCR. The mixed infections were identified in 8 patients (8/767, 1.0%). Among outpatients, the rates of rotavirus and norovirus were 30.5% and 11.6%, respectively. And 2 mixed infections were observed. Among inpatients, the positive rates of rotavirus and norovirus were 41.5% and 8.3%, respectively, in which 6 mixed infections were detected. The positive rates of rotavirus in outpatients and inpatients were analyzed by Chi-square test, which was statistically significant (*Χ*^2^ = 9.37, P = 0.002). However, the analysis of infection rates of norovirus between outpatients and inpatients was not statistically significant (*Χ*^2^ = 1.96, P = 0.161).

**Table 1:** Age distribution of rotavirus and norovirus infected cases

**Age**	**Patients n = 767**		**Outpatients n = 502**		**Inpatients n = 265**	
**Rate**	**Rotavirus(+)**	**Norovirus(+)**	**Rotavirus(+)**	**Norovirus(+)**	**Rotavirus(+)**	**Norovirus(+)**
	**263 (34.3%)**	**80 (10.4%)**	**153 (30.5%)**	**58 (11.6%)**	**110 (41.5%)**	**22 (8.3%)**
0-5 M	14 (5.3%)	5 (6.3%)	8 (5.2%)	3 (5.2%)	6 (5.5%)	2 (9.1%)
6-12 M	37 (14.1%)	20 (25.0%)	21 (13.7%)	15 (25.9%)	16 (14.5%)	5 (22.7%)
13-24 M	104 (39.5%)	26 (32.5%)	62 (40.5%)	20 (34.5%)	42 (38.2%)	6 (27.3%)
25-36 M	58 (22.1%)	19 (23.7%)	33 (21.6%)	13 (22.4%)	25 (22.7%)	6 (27.3%)
37-48 M	29 (11.0%)	8 (10.0%)	16 (10.5%)	5 (8.6%)	13 (11.8%)	3 (13.6%)
49-60 M	21 (8.0%)	2 (2.5%)	13 (8.5%)	2 (3.4%)	8 (7.3%)	0 (0.0%)
Mean age	24.2 ± 14.0	19.9 ± 15.8	24.0 ± 14.2	19.7 ± 12.8	24.5 ± 13.8	20.5 ± 13.1

Mean age is defined as mean ± standard deviation(M = month).

Higher rates of rotavirus infections were observed in children from 13 to 36 months old, and higher rates of norovirus infections were detected in children from 6 to 36 months old (Table 1). The mean age of patients positive for rotavirus was older than that for norovirus (24.2 ± 14.0 months vs. 19.9 ± 15.8 months, U = 2.19, P < 0.05). The mean ages of rotavirus and norovirus infected outpatients were 24.0 months (±14.0) and 19.7 months (±12.8), respectively, which was statistically significant (U = 2.11, P < 0.05). However, the mean ages in inpatients was not found to be statistically significant (24.5 ± 13.8 months vs. 20.5 ± 13.1 months, U = 1.30, P > 0.05).

263 rotavirus positive samples were further typed as G-serotype and P-genotype by RT-PCR. G3(40.7%) was the most prevalent G-serotype followed by G1(25.9%), G2(11.0%), G4(8.7%) and G9(1.1%). 5 G1/G3 mixtures and 2 G2/G3 mixtures were found, but 26(9.9%) samples were G nontypeable. The most common P-genotype was P[8](46.8%) followed by P[4](10.3%) and P[6](2.3%). 8 P[8]/P[4] mixed P-genotypes were identified and 99(37.6%) samples could not be typed for P-genotype. The predominant G and P combination was G3P[8](31.9%) followed by G1P[8](14.1%), G2P[4](7.2%), G1P[4](0.8%) and G2P[8](0.4%).

**Figure 1:**
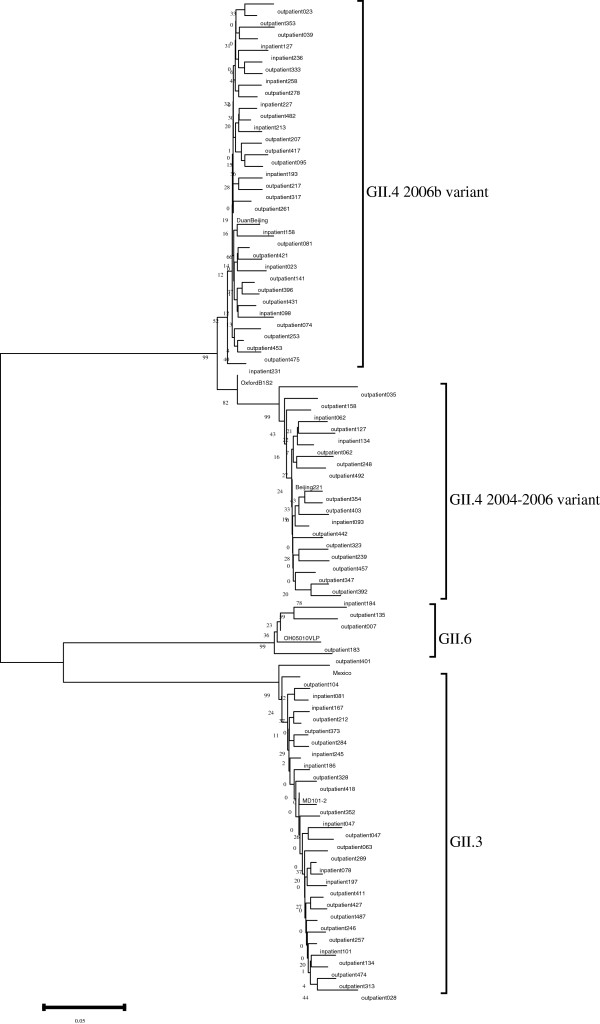
**Phylogenetic analysis based on the partial capsid sequences (282 bp).** All NoV-positive strains were genotyped. The tree was generated using the neighbor-joining method and the bootstrap values from 1000 replicates were shown on each branch. The reference strains were from NCBI GenBank: DuanBeijing(EU366113), OxfordB1S2(AY587991),Beijing221(EU839584),OH05010VLP(AB685741),Mexico(U22498),MD101-2(AY030312).

80 norovirus positive samples were genotyped for GII, and no GI strains were detected based on the capsid sequences (Figure 1). Three genotypes (GII.3, GII.4 and GII.6) were detected and GII.4 was the most prevalent genotype (61.2%) followed by GII.3 (33.8%) and GII.6 (5.0%). Phylogenetic analysis of the GII.4 sequences showed that 18 GII.4 strains belonged to GII.4 2004–2006 cluster and 31 GII.4 strains were divided into GII.4 2006b cluster.

**Figure 2:**
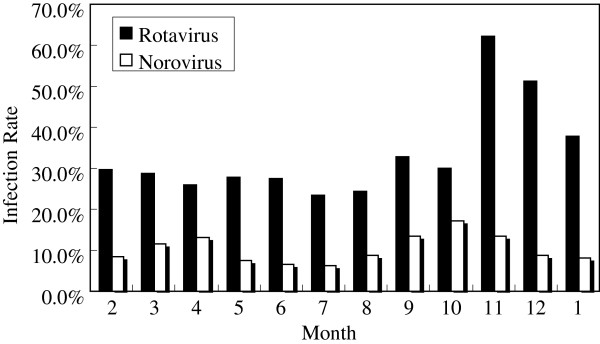
**Monthly distribution of rotavirus and norovirus infections between February 2011 and January 2012.** Rates of rotavirus infection: 29.8%; 28.8%; 25.9%; 27.9%; 27.4%; 23.4%; 24.3%; 32.8%; 30.1%; 62.2%; 51.3%; 37.9%. Rates of norovirus infection: 8.5%; 11.5%; 13.0%; 7.4%; 6.5%; 6.3%; 8.6%; 13.4%; 17.2%; 13.5%; 8.9%; 8.2%.

Infections of rotavirus and norovirus were seen throughout the year. Rotavirus peaked in the months from November to January, while norovirus infections were observed to peak from September to November (Figure 2).

The major clinical symptoms related to viral gastroenteritis in this study were acute diarrhea, vomiting and fever (Table 2). 39(14.8%) had diarrhea alone among rotavirus positive patients and 11(13.8%) were seen in norovirus infected patients. Most patients with rotavirus and norovirus experienced vomiting (88.2% and 67.5%,respectively) and fever (79.1% and 46.3%, respectively). Statistically significant rates of vomiting and fever was found between rotavirus and norovirus infections (*Χ*^2^ = 19.0 and *Χ*^2^ = 32.4, P < 0.05). No statistical significance was found in the mean frequency of diarrhea (5.08 ± 1.85 episodes/day vs. 4.99 ± 1.55 episodes/day, U = 0.43, P > 0.05) and vomiting (2.61 ± 1.35 episodes/day vs. 2.20 ± 1.47 episodes/day, U = 1.87, P > 0.05) between rotavirus and norovirus infection patients. However, the mean frequencies of diarrhea and vomiting between outpatients and inpatients for rotavirus were found to be statistically significant (U = 7.86 and U = 5.30, P < 0.05). While, the frequencies of diarrhea and vomiting between outpatients and inpatients for norovirus were also found to be significantly different (U = 3.60 and U = 2.05, P < 0.05). The mean degree of fever in rotavirus and norovirus infected patients was 38.0°C (±0.65) and 37.8°C (±0.62), respectively, which was not statistically significant (U = 1.79, P > 0.05). The degree of fever in norovirus positive cases between outpatients and inpatients was not statistically significant (U = 1.73, P > 0.05). However, there was significant difference to be observed in the degree of fever between outpatients and inpatients for rotavirus (U = 4.60, P < 0.05).

**Table 2:** Clinical manifestations of rotavirus and norovirus positive cases

**Features**	**Patients n = 767**		**Outpatients n = 502**		**Inpatients n = 265**	
	**Rotavirus(+)**	**Norovirus(+)**	**Rotavirus(+)**	**Norovirus(+)**	**Rotavirus(+)**	**Norovirus(+)**
	**263**	**80**	**153**	**58**	**110**	**22**
Diarrhea alone	39 (14.8%)	11 (13.8%)	38 (24.8%)	11 (18.9%)	1 (0.9%)	0 (0.0%)
With vomiting	232 (88.2%)	54 (67.5%)	131 (85.6%)	38 (65.5%)	101 (91.8%)	16 (72.7%)
With fever Watery stool	208 (79.1%) 53 (34.6%)	37 (46.3%) 22 (37.9%)	114 (74.5%) 48 (43.6%)	25 (43.1%) 8 (36.4%)	94 (85.5%) 101 (38.4%)	12 (54.4%) 30 (37.5%)
Diarrhea (episodes/day)	5.08 ± 1.85	4.99 ± 1.55	4.34 ± 1.12	4.55 ± 1.10	6.10 ± 2.15	6.14 ± 1.96
3-4 epi/day	124 (47.1%)	35 (43.8%)	95 (62.1%)	31 (53.4%)	29 (26.4%)	4 (18.2%)
5-6 epi/day	95 (36.1%)	37 (46.3%)	57 (37.3%)	27 (46.6%)	38 (34.5%)	10 (45.5%)
> = 7 epi/day	44 (16.7%)	8 (10.0%)	1 (0.6%)	0 (0.0%)	43 (39.1%)	8 (36.3%)
Vomiting (episodes/day)	2.61 ± 1.35	2.20 ± 1.47	2.21 ± 1.10	1.89 ± 1.13	3.16 ± 1.52	2.94 ± 1.91
1-2 epi/day	127 (54.7%)	36 (66.7%)	92 (70.2%)	27 (71.1%)	35 (34.7%)	9 (52.7%)
3-4 epi/day	85 (36.6%)	15 (27.8%)	36 (27.5%)	10 (26.3%)	49 (48.5%)	5 (37.6%)
> = 5 epi/day	20 (8.6%)	3 (5.5%)	3 (2.3%)	1 (2.6%)	17 (16.8%)	2 (12.5%)
Fever degree (°C)	38.0 ± 0.65	37.8 ± 0.62	37.9 ± 0.60	37.7 ± 0.56	38.3 ± 0.64	38.1 ± 0.70
<37.5°C	70 (33.7%)	19 (51.4%)	50 (43.9%)	14 (56.0%)	20 (21.3%)	5 (41.7%)
37.5-38.5°C	77 (37.0%)	11 (29.7%)	43 (37.7%)	8 (32.0%)	34 (36.2%)	3 (25.0%)
>38.5°C	61 (29.3%)	7 (18.9%)	21 (18.4%)	3 (12.0%)	40 (42.5%)	4 (33.3%)

## Discussion

In this study, a systematic investigation of the infection of rotavirus and norovirus was carried out in children under 5 years of age with acute diarrhea in Ji’nan, China. This study showed that rotavirus was the most common etiologic agent of acute diarrhea in children and was detected in 34.3% of patients. This finding was consistent with the previous studies in China [13,14], in which the rates of rotavirus infection were between 28% and 65%. As another common cause of viral associated diarrhea, norovirus was detected in 10.4% of 767 samples which was consistent with the results of previous surveys in China (8.9%-10.3%) [17,22,23]. However, in some other studies the rates of norovirus infection were more than 20% [24,25]. 432(56.3%) fecal samples were not detected with rotavirus and norovirus, suggesting that bacterial and parasitic pathogens or other enteric viruses such as adenovirus and astrovirus might contribute to the remaining infection [5,26,27].

Rotavirus and norovirus infections were detected in all age groups. Although the patients enrolled were under 5 years of age, 536(69.9%) stool samples were collected from children under 2 years of age. The mean age of rotavirus infected patients was 24.2 months (±14.0) and the mean age of norovirus positive patients was 19.9 months (±15.8). These findings showed that rotavirus and norovirus infections usually occurred in early childhood, which indicated children in early childhood were more susceptible to rotavirus and norovirus infection and infections might result in protective immunity against re-infection after 2 years of age.

The peak of rotavirus infections was in autumn and winter in different countries [28]. In the present study, rotavirus infections peaked from November to January. Different seasonal patterns of norovirus infections have been observed. Some studies reported a higher frequency in winter, spring or rainy seasons, whereas others showed no obvious peak season [5,17,29,30]. In this study, the higher detection rates of norovirus were observed from September to November and slightly increased infections were seen in March and April. The peak of norvirus infections appeared earlier than that of rotavirus infections in our study. However, the peak of norovirus infections obtained from other studies seemed to overlap with that of rotavirus infections [17,25].

G1-G4 rotavirus serotypes were the most common serotypes around the world and all of them were detected in the present study. G1 was considered the most common serotype worldwide [31,32]. However, serotype G3 was the predominant serotype during the period of this study in Ji’nan followed by G1, which was consistent with the previous studies in China [13,14]. G9 strains began emerging in the late 1990s and became the predominant strain in some countries. However, it was detected at a low rate in China, Japan and Korea between 0.9% and 5.9% [13,33-38]. In this study, serotype G9 was observed at a rate of 1.1%, which was similar to the previous reports. But it was much lower than that reported in some other Asian regions: 24.1% in Taiwan, 19.1% in South India and 78.3% in Malaysia [39-41].

P[8] and P[4] were the most prevalent P genotype worldwide [28,42,43]. In this study, P[8] was detected in 46.8% of rotavirus positive patients followed by P[4].

Epidemiological studies had demonstrated that the G/P combinations most frequently detected were G1P[8], G3P[8], G4P[8], G2P[4], G9P[8] and G9P[6] [44]. In China, the most prevalent combination was G1P[8] until 2000, but it had shifted to G3P[8] by 2001–2003 [14]. In our study, G3P[8] was the most common recombinant followed by G1P[8], which was similar to the results of previous studies.

Among 80 norovirus positive patients, GII.4 was the most prevalent genotype, which was consistent with the previous finding that GII.4 was the major genotype in outbreaks and sporadic acute gastroenteritis [30,45,46]. 31 GII.4 strains belonged to GII.4 2006b cluster and remaining 18 GII.4 strains clustered in 2004–2006 variant. The results suggested that there was no new GII.4 strain to emerge in this region since the GII.4 2006b strains were detected in Europe in 2005–2006.

In this study, we detected typical symptoms for rotavirus and norovirus infections including diarrhea, vomiting and fever. Among patients with rotavirus infection, vomiting was more common than fever and watery stool, which was similar to the results from children with norovirus infection. Rotavirus infections resulted in a higher rate of vomiting and fever than that of norovirus infection. However, the presence of watery stool was at a similar level. Comparison of the frequency of diarrhea and vomiting and the degree of fever between rotavirus and norovirus infections, there was no significant difference to find. Among 80 patients with norovirus infection, 54(67.5%) had vomiting and 37(46.3%) had fever, which was consistent with a previous study in China [25]. However, the rates of vomiting and fever from a survey carried out in adults in Beijing were 27.1% and 2.1% respectively, which were lower than that from our study. These findings indicated that norovirus infection resulted in a higher rate of vomiting and fever in children than in adults.

## Conclusions

In conclusion, the findings of this study showed that rotavirus and norovirus were the important pathogen in childhood diarrhea in Ji’nan, China. Infections mainly occurred under 2 years of age and peaked in autumn and winter. The common clinical symptoms were diarrhea, vomiting and fever. Although there was no death case to report, the infection still posed a serious threat to the health of children. This study provided useful data for epidemiologic and clinical features of rotavirus and norovirus infections, which would provide scientific support for the development of effective vaccines to reduce the morbidity and mortality.

## Materials and methods

### Fecal specimen collection

A total of 767 fecal specimens were collected from children under 5 years of age (502 outpatients and 265 inpatients) with acute diarrhea between February 2011 and January 2012 at Shandong University Qilu Hospital and Qilu Children’s Hospital in Ji’nan, China. Acute diarrhea was defined as the presence of three or more episodes of watery or looser than normal stool in a 24-hour period. Patients enrolled met the following inclusion criteria: (1) consultation at the hospital clinics or hospital admission for acute diarrhea; (2) absence of leukocytes in the stool by microscopic examination; (3) absence of blood and pus from the stool. Simultaneously, the clinical information from these children was collected from their parents who were asked to sign an informed consent. The stool samples were frozen and stored at -20°C for processing later. This study was approved by the Ethics Committee of Shandong University Qilu Hospital.

### Viral RNA extraction

A 20% (w/v) suspension of each stool sample was prepared and centrifuged at 1,500 g for 20 min. A 200 μl-supernatant was taken from suspension for RNA extraction using the QIAamp Viral RNA Mini Kit according to the manufacturer’s instructions. RNA suspension were stored at -70°C until used.

### Detection of rotavirus

Stool specimens were tested for rotavirus by the Accudiag™ Rotavirus (Fecal) ELISA Kit. The sensitivity of this kit is 100% and the specificity is 97.1%. 100 μl of stool suspension was added to the microwell and incubated for 30 min at room temperature. The rotavirus specific polyclonal antibodies attached to the well could capture the rotavirus antigens present in the suspension. Additional antibodies conjugated to horseradish peroxidase were added and incubated for 5 min at room temperature. These antibodies could sandwich the antigens. After washing to remove unbound enzyme, a chromogen was added and incubated for 5 min at room temperature. Then, 100 μl of sulfuric acid was added to end the reaction. Optical density (OD) value was measured at 450 nm. Absorbance reading of 0.15 OD and above indicated the sample contained rotavirus antigen.

Rotavirus positive specimens were further characterized for G and P type by RT-PCR with type-specific primers (Table 3). RT-PCR for typing VP7 gene was carried out using primers specific for G1-G4 and G9. The reaction of reverse transcription (RT) was performed with one cycle at 45°C for 30 min, followed by 35 cycles of PCR: at 94°C for 30 sec, 48°C for 30 sec, 72°C for 1 min and a final extension at 72°C for 7 min. RT-PCR for typing VP4 gene was performed using primers specific for P[8], P[4] and P[6]. The steps of RT-PCR were same as described in VP7genotyped.

**Table 3:** Primers used in genotyping for rotavirus

**Primers**	**G/P typing**	**Sequence(5’-3’)**	**Product (bp)**
9Con1	G(+)	TAGCTCCTTTTAATGTATGG	-
9Con2	G(-)	GTATAAAATACTTGCCACCA	905
9 T1	G1(-)	TCTTGTCAAAGCAAATAATG	159
9 T2	G2(-)	GTTAGAAATGATTCTCCACT	245
9 T3	G3(-)	GTCCAGTTGCAGTGTTAGC	467
9 T4	G4(-)	GGGTCGATGGAAAATTCT	404
9T9B	G9(-)	TATAAAGTCCATTGCAC	111
4Con3	P(+)	TGGCTTCGCCATTTTATAGACA	-
4Con2	P(-)	ATTTCGGACCATTTATAACC	877
1 T1	P[8](-)	TCTACTTGGATAACGTGC	346
2 T1	P[4](-)	CTATTGTTAGAGGTTAGAGTC	484
3 T1	P[6](-)	TGTTGATTAGTTGGATTCAA	268

The PCR products were resolved on 1.5% agarose gels, stained with ethidium bromide and visualized under UV light to determine the G and P type.

Negative specimens for rotavirus tested by Elisa kit were selected at random (252 samples) to confirm the presence of rotavirus.

### Detection of norovirus

An RT-PCR was carried out for norovirus genome amplification using primers GI-SKF(5′-CTGCCCGAATTYGTAAATGA-3′)/GI-SKR(5′-CCAACCCARCCATTRTACA-3′)and COG2F(5′-CARGARBCNATGTTYAGRTGGATGAG-3′)/G2-SKR(5′-CCRCCNGCATRHCCRTTRTACAT-3′). RT was performed at 50°C for 1 h using a random primer and SuperScriptII reverse transcriptase. PCR was performed under the following steps: 94°C for 3 min, 40 amplification cycles at 94°C for 1 min, 55°C for 30 sec, 72°C for 60 sec and a final extension at 72°C for 7 min.

PCR products were resolved on 1.5% agarose gels, stained with ethidium bromide and visualized under UV light, then purified using the QIAquik PCR Purification Kit.

Amplification products were sequenced using a Big-Dye Terminator Cycle Sequencing Kit and an ABI 3730XL DNA Analyzer. The obtained sequences were aligned by Clustal X (version 2.0) followed by phylogenetic analysis using MEGA (version 5.0). The phylogenetic tree was generated using neighbor-joining method with 1000 bootstrap replicates.

To confirm the true negatives for norovirus, all stool samples were tested using ProSpecT™ Norovirus Microplate Assay.

The nucleotide sequences from this study were deposited in GenBank under accession numbers: KC999725—KC999804.

### Statistical analysis

Data was analyzed using SPSS16.0. The results were shown as proportion and the mean value with standard deviation (SD). Chi-square and *U* test were used to compare characteristics of each group. For all analysis, statistical significance was defined as P values less than 0.05.

## Abbreviations

ORF: Open reading frame; ELISA: Enzyme-linked immunosorbent assay; OD: Optical density; SD: Standard deviation.

## Competing interests

The authors declare that they have no competing interests.

## Authors’ contributions

LS performed the experiments, participated in the design of the study and wrote the initial draft of the manuscript. JS, LS, SC and HL helped to carry out the experiments and to analyze the data. LM supervised, helped to design the study and finally edited the manuscript. All authors read and approved the final manuscript.
